# Engineered Reactive Interfaces Enable Mass Spectrometry Imaging of Multiple Thiols for Decoding PFOS-Induced Redox Dysregulation

**DOI:** 10.1021/acs.analchem.5c05993

**Published:** 2025-12-29

**Authors:** Hongmei Xu, Thomas Ka-Yam Lam, Simin Zhang, Lei Guo, Chris Kong Chu Wong, Chuan Dong, Zongwei Cai

**Affiliations:** † Institute of Environmental Science, 12441 Shanxi University, Taiyuan 030006, China; ‡ State Key Laboratory of Environmental and Biological Analysis, Department of Chemistry, 26679 Hong Kong Baptist University, Kowloon 999077, Hong Kong SAR, China; § Eastern Institute of Technology, Ningbo 315200, China; ∥ Croucher Institute for Environmental Sciences, Department of Biology, Hong Kong Baptist University, Kowloon 999077, Hong Kong SAR, China

## Abstract

Spatial profiling of multiple thiols shows great significance in elucidating the redox status across tissue microregions and understanding the molecular mechanisms of oxidative stress injury. Traditional matrix-assisted laser desorption/ ionization mass spectrometry imaging (MALDI MSI) relies on chemical derivatization for thiol visualization, but multistep derivatization protocols and nonspecific matrix-adduct formation compromise both detection sensitivity and spatial mapping fidelity. Herein, we engineer a reactive interface-assisted chemical derivatization platform for sensitive assessment of multiple thiols in various tissues via forming the “matrix-tissue-interface” sandwich structure. Reactive interface that predeposited with *N*-(9-Acridinyl) maleimide (NAM) probes enables profile multiple thiols including cysteamine (MEA), cysteine (Cys), cysteinyl-glycine (Cys–Gly), glutathione (GSH), and ergothioneine (ET) across various tissues. The increased sensitivity is likely due to the accelerated reaction efficiency that arises from the locally high NAM concentrations in the tissue–NAM interface, coupled with the sandwich architecture that mitigates ion suppression of NAM probes and prevents matrix–NAM interaction. The results demonstrated distinct tissue-specific distribution patterns of various thiols as well as redox dysregulation of kidney induced by PFOS exposure. This innovative MSI methodology offers a robust route to enhance the derivatization performance for low-abundance molecule imaging, facilitating the investigation of oxidative stress-related disease mechanisms and the toxicological effects of pollutant.

## Graphical abstract

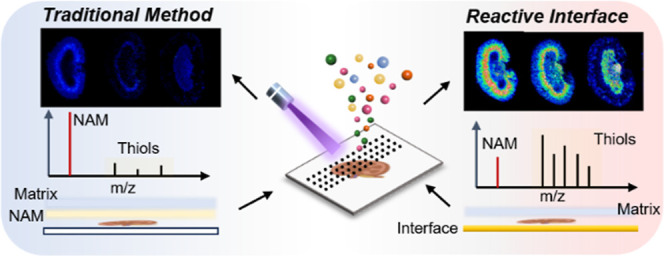

## Introduction

Biological thiols, such as Cys, Cys–Gly, and GSH, not only serve as integral components of the antioxidant defense system that safeguard cells against oxidative damage but also as neuroactive agents that modulate metabolic processes and maintain physiological equilibrium.^1–3^ Glutathione deficiency is clinically associated with elevated risks of cancer, Alzheimer’s disease, and hepatic injury.^4,5^ As the rate-limiting substrate for GSH synthesis, cysteine demonstrates independent therapeutic potential in nutritional supplementation.^6,7^ In addition, most thiols engage in dynamic thiol–disulfide exchange, constructing interconnected metabolic networks that combat oxidative stress through upstream-downstream pathway coordination and mutual reinforcement.^8,9^ Consequently, the concurrent detection and differentiation of these thiols within metabolic pathways are essential for unraveling oxidative stress signaling cascades and understanding the etiology of diseases.

Currently, significant efforts have focused on sensitive techniques for profiling of multiple thiols.^10–12^ Among them, fluorometric methods leverage tailored molecular probes that react specifically with sulfhydryl (-SH) groups, enabling the sensitive identification of thiol species.^13–16^ However, due to analogous thiol reactivity, the differentiation of individual species using fluorescent probes typically relies on complex reactive moieties. Moreover, spectral overlap imposes significant constraints on the multiplexing capabilities of these assays.^17,18^ In comparison, mass spectrometry offers unique advantages due to its inherent ability of label-free detection, allowing distinguish multiple thiols by their molecular weight tags.^19^ Liquid chromatography–mass spectrometry (LC–MS) stands as the mainstream method for thiol detection and enables quantitative assessment of multiple thiols at tissue and organism levels.^20–22^ However, LC–MS can damage tissue structural integrity and lacks the ability to preserve and reveal the spatial distribution of thiols, which is crucial for understanding redox compartmentalization and its pathological implications.^23–25^

Mass spectrometry imaging (MSI) techniques are now being developed to bridge this analytical gap, which allows untargeted evaluation of analytes and their spatial distribution with a label-free manner in a single experimental procedure.^26,27^ Matrix-assisted laser desorption/ionization (MALDI) remains the predominant ionization technique for MSI, valued for its accessibility and advancements in spatial resolution.^28,29^ However, effective thiol visualization is hindered by poor instability, low physiological concentrations, and interference from endogenous compounds.^30^ On tissue chemical derivatization (OTCD) has been adopted to improve MALDI MSI interrogation of thiols by introducing precharged modules to facilitate desorption/ionization efficiency of targets.^31–35^ The fluorescent reagent *N*-(9-acridinyl)­maleimide (NAM) probe has been extensively employed in fluorescence assays and LC–MS analysis of thiols owing to its exceptional reaction efficiency and thiol-specific selectivity via Michael addition reaction.^36,37^ In the recent advance, structurally analogous probes were engineered gradually and applied in MALDI MSI analysis of multithiol dysregulation by integrating with chemical derivatization strategy.^33,34^ In this process, derivatization reagents were sprayed on the surface of tissues by using hand-held or automated pneumatic sprayers. To enhance the reaction efficiency with target molecules, the reagents are applied iteratively under carefully optimized conditions, thus inadvertently diminishing detection sensitivity due to ion suppression effects from the derivatization reagents.^38^ Moreover, interactions between commercial matrices and derivatization reagents may impair matrix crystallization, leading to a reduced detection sensitivity and compromised spatial resolution of MSI.

To overcome the sensitivity limitation in OTCD-based MALDI-MSI, we engineered a reactive interface-assisted chemical derivatization (RICD) platform for imaging of multiple thiols within biological tissues (Scheme 1). The reaction interface was engineered through precise deposition of permanently charged maleimide probe (NAM) onto indium tin oxide (ITO) substrates for reacting with thiols. By further throwing tissues and depositing a commercial matrix, the “matrix-tissue-interface” sandwich structure was constructed and the distribution of thiols in various mouse tissues was profiled via MALDI MSI (Scheme 1a). In comparison to conventional tissue derivatization techniques, the RICD method exhibited markedly improved sensitivity for the visualization of thiols such as MEA, Cys, GSH, Cys–Gly, and ET across diverse tissues, even achieving up to 19-fold signal enhancement in Cys–Gly assessment. Furthermore, it efficiently avoided the delocalization of the products and the ion suppression of derivatization reagents for accurate thiols mapping. The RICD-based MSI approach enabled profiling of multiple thiols in mouse kidney, brain, heart, liver, and spleen tissues and also assessed the altered redox state in mouse kidney tissue under PFOS exposure (Scheme 1b). The RICD-mediated imaging principle creates a new paradigm in mass spectrometry derivatization imaging, thus facilitating the in-depth understanding of redox networks related to the pathological mechanisms of diseases as well as the toxicological effects of pollutants.

**Scheme 1:**
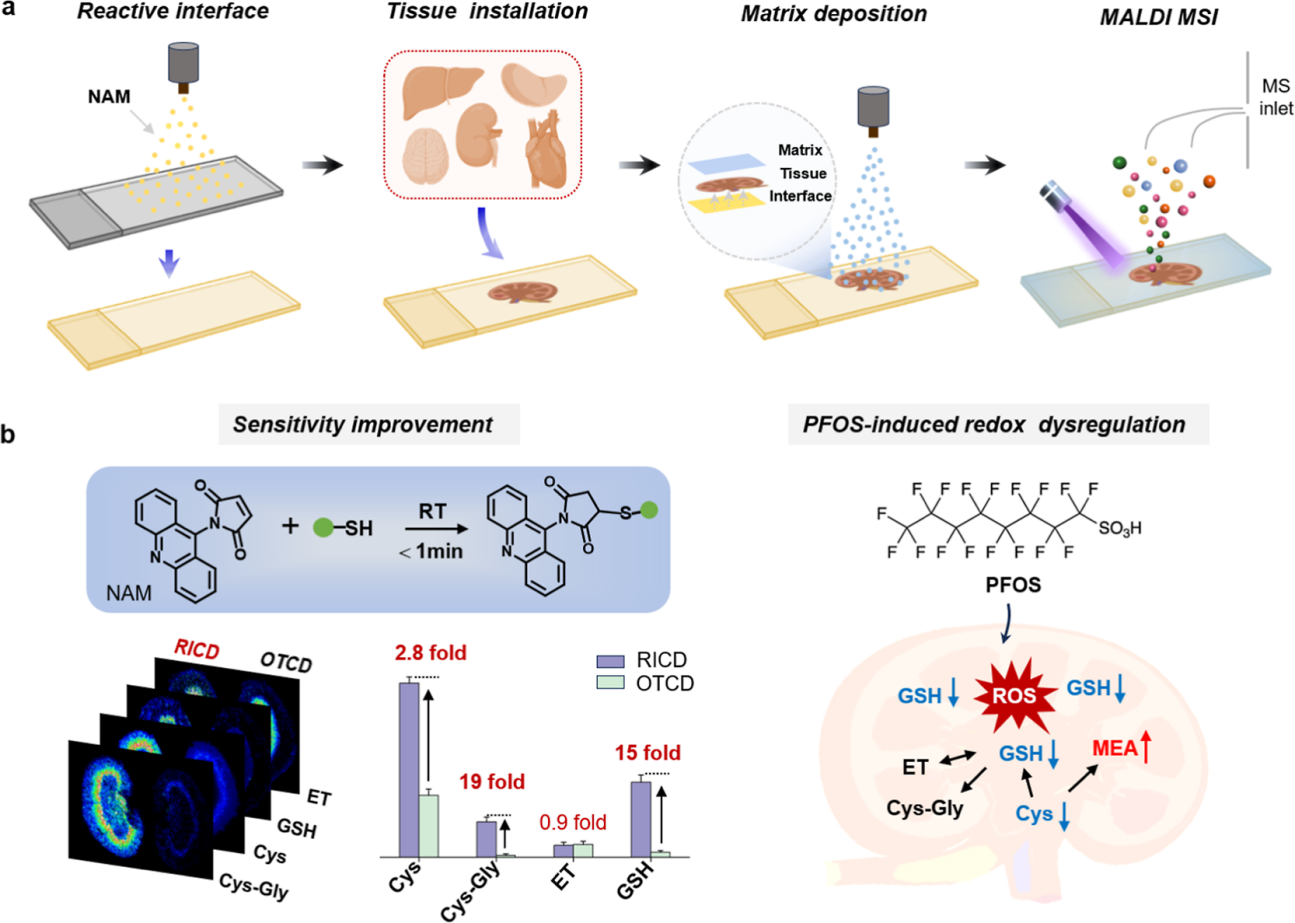
Schematic Illustration of the Reactive Interface-Assisted MSI Platform for Imaging of Multiple Thiols in Various Tissues: (a) Representative Process of MALDI MSI of Thiols based on RICD; (b) RICD-Based MSI of Thiols with Improved Sensitivity, as well as the Application in Underlying the Redox Dysregulation Induced by PFOS Exposure; RICD: Reactive Interface-Assisted Chemical Derivatization, OTCD: On Tissue Chemical Derivatization

## Experimental Section

### NAM Probes-Based In Vitro Assessment of Thiols

As for in vitro assessment of thiols, 10 μL of standard thiol solution with different concentrations was added into 180 μL of an aqueous solution containing 10 μL of NAM probes (5 mM in 70% ACN). After reacting at room temperature for 30 min, 1 μL of reaction solution was mixed with 1 μL of DHB solution (20 mg/mL in 70% ACN, 1% H_3_PO_4_) and deposited on the 384-well stainless steel MALDI plate. As for quantification of thiols in tissue extracts, the reserpine was selected as the internal standard, and 0.5 μL of reaction solution and 0.5 μL of internal standard were mixed with 1 μL of DHB matrix before analysis. The selectivity of NAM probes was confirmed by incubating NAM probes with various interferents including lysine, arginine, and threonine at room temperature for 30 min.

### Reactive Interface-Mediated Derivatization of Thiols in Tissues

The reactive interface was prepared as follows: NAM solution (5 mM in 70% ACN) was sprayed onto the conductive slide of ITO using a TM-Sprayer (HTX Technologies, Carrboro, NC) with a flow rate of 30 μL per minute. The nozzle nitrogen gas pressure and temperature were set to 10 psi and 60 °C, respectively. The nozzle height, track speed, track spacing, and drying time were set to 4 cm, 1200 mm/min, 2.5 mm, and 10 s, respectively. To optimize the NAM deposition concentration, 3, 6, 10, 15, and 20 spray passes were applied. Frozen tissues were sliced into 10 μm sections using a CryoStar Nx70 cryostat (Thermal Fisher Scientific, Walldorf, Germany) and then were thaw-mounted on the NAM-predeposited ITO glass slides. The reactive interface was incubated in a humid environment at room temperature for 2 h and then dried under vacuum for 60 min before matrix application. After these procedures, matrix DHB (20 mg/mL in 90% MeOH containing 0.1% FA) was deposited at a flow rate of 50 μL per minute and a speed of 1200 mm/min. The nozzle temperature was set to 70 °C, and other parameters were consistent with those of the NAM deposition procedure.

In comparison, the traditional derivatization process was also performed. In brief, the tissue sections were thaw-mounted on an ITO glass slide, which was further deposited with NAM probes. The derivatization condition was the same as the NAM-predeposited procedures. After being sprayed with the NAM probe, the tissue sections were also incubated on a humid environment at room temperature for 2 h. In final, the tissue sections were dried in a vacuum desiccator for 1 h prior to matrix deposition. The step of DHB deposition was the same as that described above.

### MSI Data Processing and Analysis

The MSI experiment was performed using a timsTOF fleX MALDI 2 (Bruker Daltonics) equipped with a SmartBeam 3D laser (355 nm wavelength for MALDI). The laser was set to a 65% laser power with a laser frequency of 10,000 Hz and 150 laser shots per pixel. The detection range was set to 100–1200 *m*/*z* in the positive mode, and the lateral resolution was 50 μm in the single mode for each pixel analysis. Instrument calibration was conducted using an ESI-L low-concentration tuning mix (Agilent Technologies, U.S.A.). The products underwent initial filtration through comparison with untreated tissues, and the precise mass of thiols was determined by subtracting the mass of the NAM reagent (M-C_17_H_10_N_2_O_2_). Identification of the thiols was achieved by querying the accurate mass against the Human Metabolome Database (HMDB) with a mass error of less than 5 ppm and referencing the pertinent literature. Subsequent validation of the derivatized products was conducted by matching their tandem mass spectra with those of authentic standards. The MS/MS analysis was performed using a collision energy range of 10–40 eV with an isolation width of 1 Da. Images were generated from SCiLS Lab MVS version 2023b Premium 3D (Bruker Daltonics, Germany) and normalized to the root-mean-square with a mass tolerance of 15 ppm.

## Results and Discussion

### Evaluation of Reactivity and Specificity of NAM Probes

Direct MS inquiry of thiols is challenging due to their poor ionization efficiency and ease of oxidation. The introduction of derivatization reagents equipped with remarkable reactive sites to thiols and precharged moiety for enhanced ionization efficiency can improve the MS responses. Here, we selected a commercial fluorescent labeling reagent NAM as the derivatization probe for constructing the reactive interface that enabled MS visualization of thiols with improved sensitivity. In this probe, maleimide was in charge of reacting with sulfhydryl group of targets via the Michael addition reaction, which presented obvious advantages including fast reaction rate, moderate reaction condition, and specific reactivity. In addition, acridinyl fragment boosted the ionization efficiency of products due to its strong UV absorption at 355 nm, consistent with the MALDI laser wavelength range for incremental energy transformation efficiency (Figure S1). The reactivity between NAM probe and standard thiols including MEA, Cys, Cys–Gly, ET, and GSH was investigated first. As shown in Figure 1a,b, different thiols also produced differentiable *m*/*z* values that were the product tags, thus allowing multiplex accessing of thiols in a single MS inquiry. Considering the potential interference of other metabolites reacted with NAM probes, the specificity was corroborated by incubating NAM probes with GSH and other interferents (including lysine, arginine, and threonine), respectively. The results indicated that NAM probes reacted only with GSH, and the relative intensity of product *I*_product_/*I*_IS_ (using reserpine as the internal standard) increased with incremental concentrations of GSH ranging from 10 μM to 2 mM, exhibiting a linear relationship *I*_Product_/*I*_IS_ = 0.161 X + 0.017, with the detection limit of 1.22 μM (Figures 1c,d and S2). In addition, the reaction efficiency between the NAM probe and thiols was also evaluated with adoption of GSH as the model molecules. Upon the addition of the NAM probe to the GSH solution at room temperature, the reaction initiated instantaneously and reached a maximum within 30 min. The superior reaction rate of NAM probe not only improves the derivatization efficiency but also effectively avoids the occurrence of the thiols autoxidation in tissues (Figure 1e).

**Figure 1:**
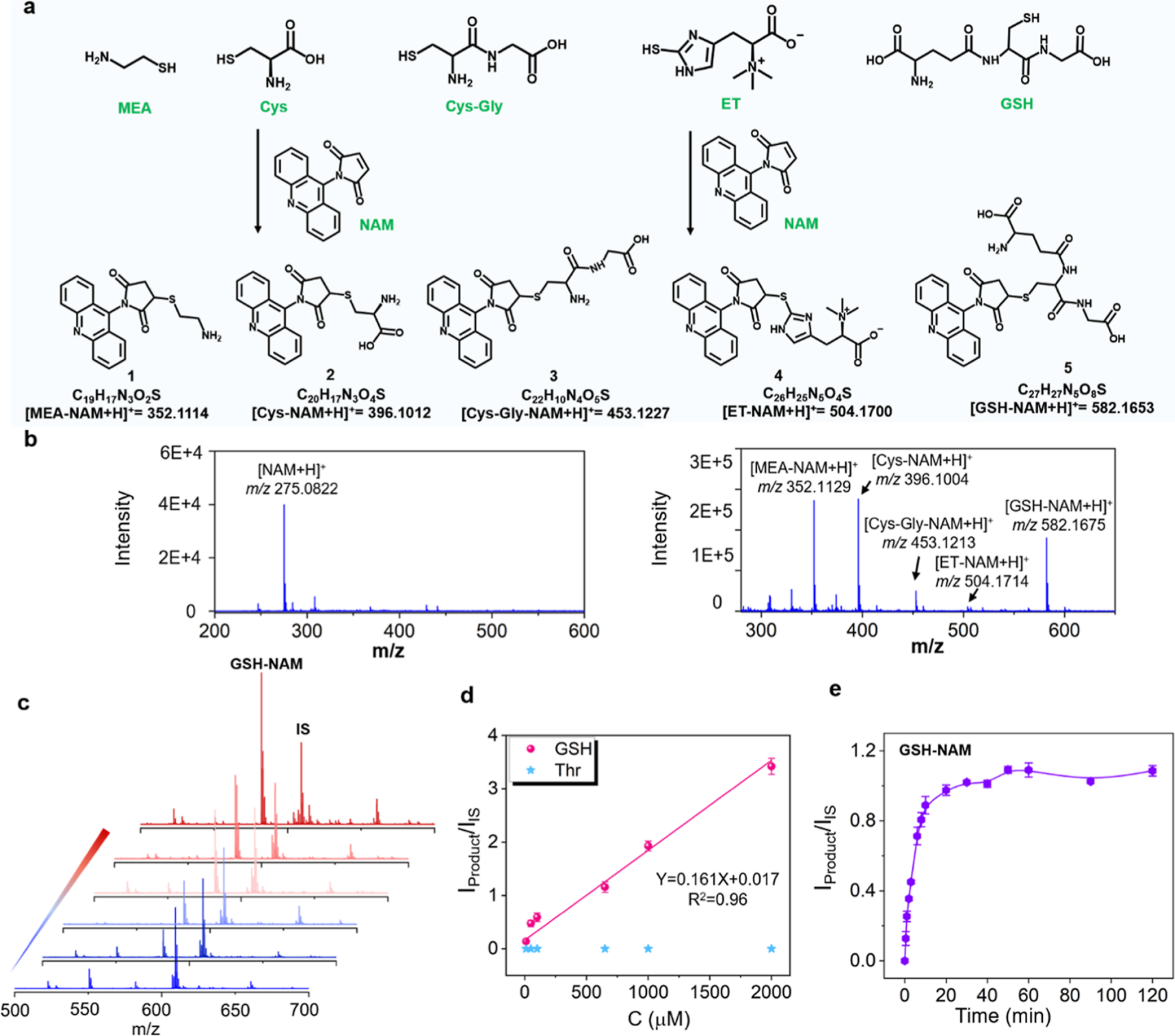
(a) Chemical reaction of NAM probe with the free thiols to form precharged products. (b) MALDI MS spectra of NAM probe and the products. (c) MALDI MS spectra of product of the NAM probe with different concentrations of GSH (from bottom to top: 5 μM, 10 μM, 0.1 mM, 0.2 mM, 0.65 mM, and 1 mM). (d) Relationship between the intensity ratio of product (GSH-NAM or Thr-NAM) and the concentrations of the GSH or Thr. The reserpine was selected as the internal standard (*m*/*z* 609.2806). *I*_product_ and *I*_IS_ represented MS peak intensity of product and IS. (e) Relative intensity of GSH-NAM varied with different incubation time.

### NAM Probe as the Reactive Matrix for MSI

Beyond its role as a reactive probe for thiol derivatization, NAM also served as a matrix to facilitate the desorption and ionization of target molecules. A comparative analysis of signal intensities of products was performed using commercially available matrices, including DHB, CHCA, and the NAM probe (refer to Figure S3a). DHB and CHCA exhibited superior detection efficiencies for a majority of thiols, such as MEA, Cys, and GSH,- however, they were ineffective in detecting ET. Conversely, the NAM probe demonstrated distinctive ionization-enhancing capabilities for ET, likely due to structural compatibility and strengthened matrix–analyte interactions via π–π stacking. Importantly, the efficacy of the NAM probe as a matrix was further corroborated by its successful application in the spatial mapping and characterization of lipids and metabolites within tissue sections (refer to Figure S3b). Despite these promising findings, the performance of NAM probe was somewhat inferior to that of conventional matrices, necessitating the continued use of DHB as the matrix for imaging purposes.

### Reactive Interface-Mediated Sensitivity Enhancement for Spatial Profiling of Thiols in Tissues

The reactive interface adorned with NAM probes is hypothesized to exhibit enhanced sensitivity over conventional tissue derivatization techniques for profiling thiols within biological tissues. To confirm this, we compared the performance of conventional derivatization methods with our proposed method in terms of target molecule detection sensitivity, respectively. The intense NAM peak (*m*/*z* 275.0813) arising from reagent accumulation in the spraying process was observed using conventional derivatization techniques, which significantly obscured colocalized biomolecular signatures (Figure 2a,b). In contrast, our reactive interface system demonstrated a 65% reduction in NAM-derived background interference, with the advantage in access to low-abundance species, and in particular, about 72% of metabolites exhibited increased signal intensity (Figure S4). In addition, the reactive interface could profile multiple thiols with enhanced sensitivity and satisfactory spatial resolution, whereas conventional derivatization methods only detected ET under the same condition. Especially for low-abundance targets such as Cys, Cys–Gly, and GSH, the product signal intensity was enhanced by 2.8-fold, 19-fold, and 15-fold, respectively, thus demonstrating our superior capability in the comprehensive mapping of the metabolic network of thiols in tissues (Figure 2c,d). The heightened detection sensitivity may be ascribed to several reasons. First, compared to traditional OTCD, precoating of NAM probes to establish the rough reaction interface can maximize the NAM utilization efficiency, producing locally high NAM concentrations (with the 5.4-fold intensity enhancement) on our reactive interface, which may accelerate the diffusion of the NAM probes into the tissues under humid conditions, thus expediting the reaction efficiency (as shown in Figures S5 and S6). In addition, the “matrix-tissue-interface” sandwich structure physically segregates ionization processes of NAM probes by tissue barrier, thereby mitigating its ion suppression effects and preventing its interaction with the DHB matrix, concurrently improving matrix crystal homogeneity (as shown in Figure S7).

**Figure 2:**
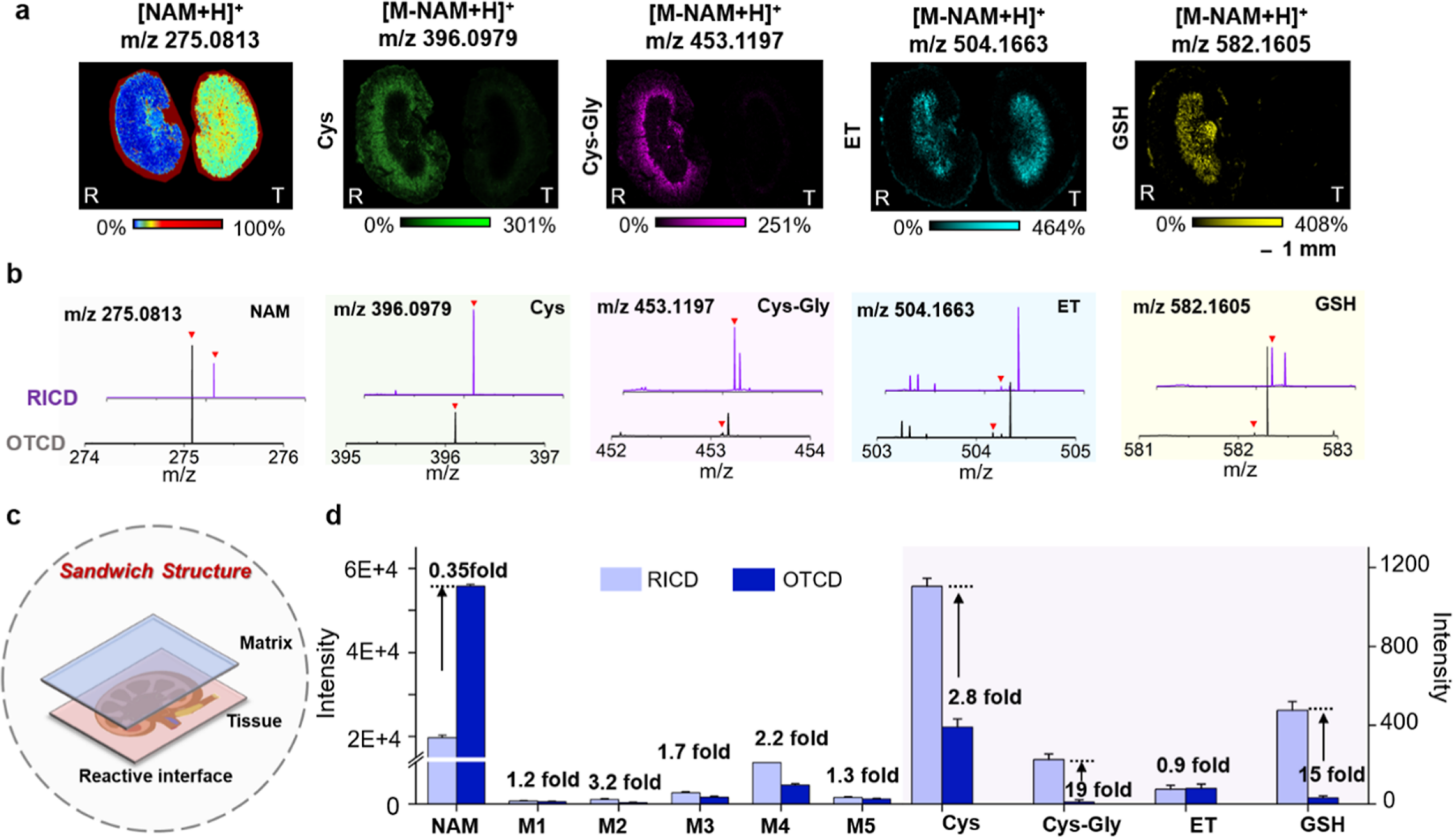
Comparison of the derivatization efficiency of thiols assisted by the traditional method and reactive interface. (a,b) Spatial distribution and representative MALDI MS spectra of products in kidney using the traditional derivatization method and reactive interface. R: RICD method, T: traditional OTCD method. (c) Schematic diagram of reactive interface-mediated sensitive detection of thiols via the construction of sandwich structure. (d) Fold changes visualization between traditional derivatization method and reactive interface for representative metabolites and thiols. The intensity and error bar represented the mean intensity of three biological replicates and related SD. The intensity of products was the whole-section mean intensity across the entire tissue.

In order to maximize the reaction efficiency, we investigated the effects of various factors, including the coating concentrations of NAM probes in the reactive interface, tissue slice thickness, and incubation duration on the reaction efficiency, respectively. Given the potential influence of tissue thickness on the diffusion of NAM probes, we evaluated the detection sensitivity across varying tissue thicknesses, specifically 7 μm, 10 μm, and 14 μm. As depicted in Figure S8, the product peaks including Cys, Cys–Gly, ET, and GSH were distinctly observed in both 7 and 10 μm tissue sections. However, due to the inherent instability associated with obtaining a 7 μm tissue section, we adopted a 10 μm section for subsequent experiments. In addition, the concentrations of the NAM probes coated on the surface of the ITO glass slides also greatly affect the reaction efficiency. Through systematic optimization, we found that the intensity of the products initially increased with the probe concentrations but subsequently declined, likely due to suppression of ionization at higher probe levels. Therefore, we selected the NAM dosage of 0.039 mg/cm^2^ as the optimal condition (Figure S9). Further optimization of the incubation time between tissue sections and the reactive interface under humidified conditions demonstrated progressive improvement in the reaction efficiency with prolonged incubation time (Figure S10). Based on this, 2 h incubation period was established as the standard protocol.

### Evaluating the Accuracy of Reactive Interface-Based MSI

Analyst delocalization is the most common problem in MSI, especially during the derivatization process, which leads to the distortion of spatial distribution information on analytes and thus affects the accurate profiling of tissue microregions by MSI.^39^ In our experiments, the reactive interface that precoated with derivatization reagents can circumvent metabolite migration, which is typically induced by the repetitive spraying of derivatization reagents and matrix in traditional derivatization protocols. Therefore, the precision of our method in profiling the spatial distribution of metabolites was evaluated first. Initially, we selected GSH as a model compound to compare the concordance of its distribution in the kidneys between our method and traditional MSI techniques (using NEDC as the matrix). As depicted in Figure 3a, our approach revealed that GSH predominantly localized in the renal pelvic region of kidney, maintaining superior imaging resolution and in keeping with the distribution from conventional MSI results. Furthermore, we examined the distribution patterns of other metabolites with specific distribution characteristics by using our method. The ion signal at *m*/*z* 568.3363, *m*/*z* 482.3571, *m*/*z* 338.1152, and *m*/*z* 258.1099 were distributed in outer cortex, inner cortex, medulla, and renal pelvis, respectively, consistent with segmentation regions, thus indicated that our technique accurately delineated the distribution of these metabolites without compromising imaging quality (Figure S11 and Table S1). Comparable results were also observed in assessing distribution profiles of representative molecules within brain tissue (Figure 3b). Especially, some metabolite ions presented enhanced intensity by our method, further demonstrating the role of the MS matrix of the NAM probes. These results demonstrated that our method achieved an accurate assessment of metabolite distribution with improved detection sensitivity via acting as the assistant matrix.

**Figure 3:**
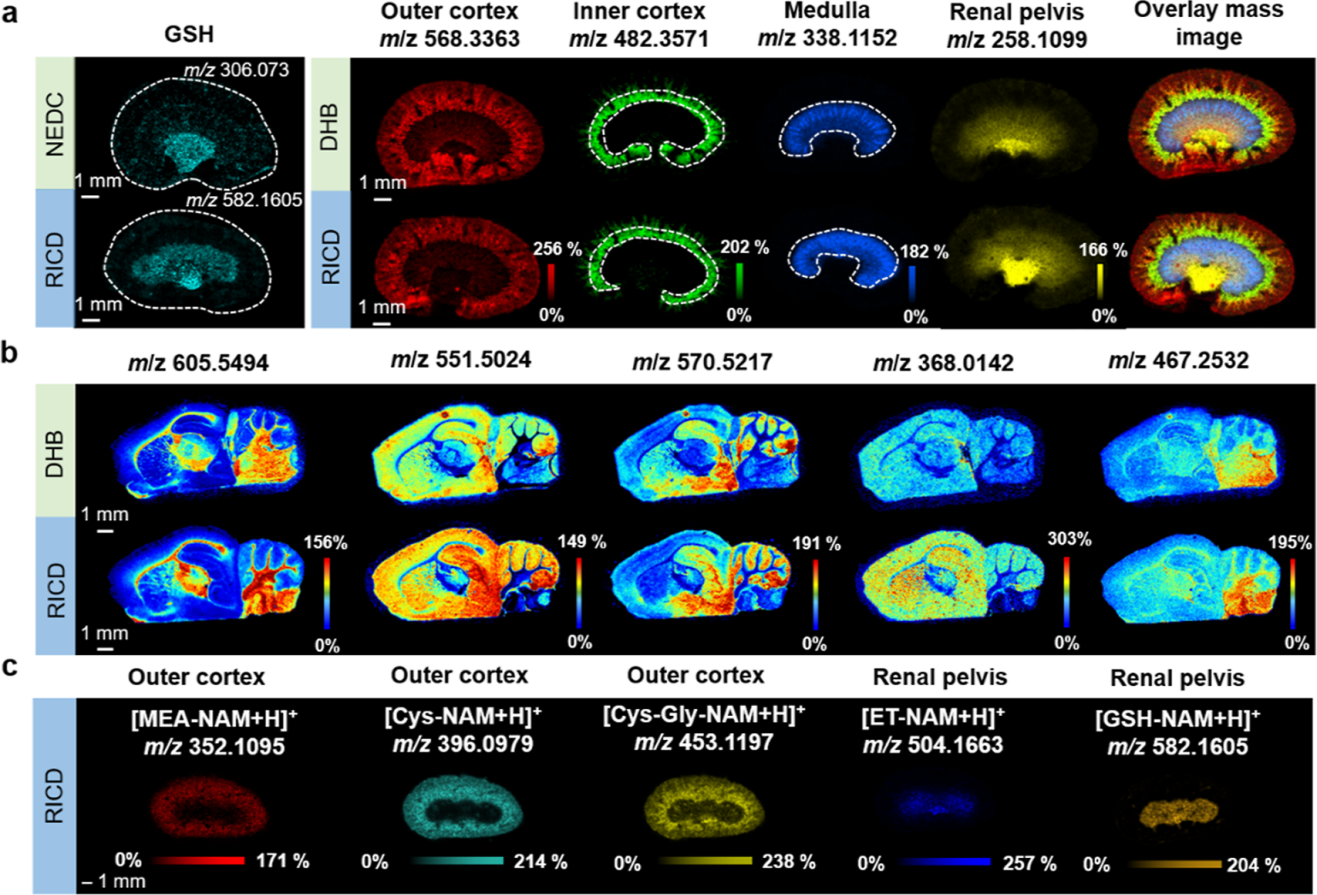
Evaluation of the accuracy of reactive interface-based MSI results. Comparison of the distribution of GSH and representative metabolites in kidney (a) and brain (b) using the traditional MSI method and our reactive interface. (c) RICD-mediated spatial distribution of thiols in kidney.

Under the optimized experimental conditions, a single session of MSI was capable of capturing the distribution of various thiols with different adducts, including MEA, Cys, Cys–Gly, ET, and GSH in the kidney (as shown in Figures 3c, S12, and Table S2). As key regulators of redox processes, GSH and ET were found to be predominantly localized in the renal pelvic region, whereas MEA, Cys, and Cys–Gly were in the outer cortex region. In comparison, direct MSI was limited to providing distribution information on ET or GSH alone by using DHB and NEDC matrices (Figure S13), loss of the location of other low-abundance thiols, thus highlighting the superior capability of our method in comprehensively mapping the distribution microregions of thiols in tissues.

### Spatially Profiling of Thiols in Different Tissues

The interconversion of different thiols such as Cys, Cys–Gly, and GSH is tightly regulated and varies across tissues based on their metabolic demands and physiological roles.^40^ Understanding their distribution and metabolic relationships provides insights into tissue-specific redox regulation and the pathophysiology of diseases related to oxidative stress. Herein, we demonstrate its capability for simultaneous imaging of multiple thiols across various tissues. The protonated peak [M + H]^+^ was selected as the quantitative indictor due to the dominated intensity and the consistent variation of other adducts within various tissues (Figure S14). As depicted in Figure 4, a significant number of thiols including MEA, Cys, Cys–Gly, ET, and GSH, within distinct tissues such as the heart, liver, spleen, kidney, and brain were profiled simultaneously. The liver dominated GSH synthesis and showed highest concentration of GSH due to its potential in detoxification and antioxidant defense.^41^ GSH also showed a higher signal intensity in the brain to protect neurons and glial cells from oxidative damage and maintain neurotransmitter balance. A significant level of Cys–Gly and Cys was observed in kidney after decoding by MALDI MSI. It could be attributed to the high γ-glutamyl transferase (GGT) enzyme activity in kidney to promote the cleavage of GSH into glutamate and Cys–Gly, which was further hydrolyzed into Cys and glycine by dipeptidases.^42^ In addition, the presence of Cys–Gly in the brain was indicative of the ongoing turnover and synthesis of GSH to combat oxidative stress. MEA served as a key intermediate in the metabolic pathway of Cys, and as a result, the kidney and liver, which were the primary organs responsible for Cys metabolism and synthesis, exhibited higher concentrations of MEA. ET acted as a potent scavenger of ROS, functioning both as an efficient antioxidant and a mediator of cellular antioxidant defense system, thereby influencing the cellular GSH redox balance.^43,44^ The significant accumulation of ET in the liver, kidney, and spleen was observed and primarily attributed to the high metabolic activity and exposure to oxidative stress in these tissues, necessitating ET with antioxidant and cytoprotective properties to preserve their regular operation.

**Figure 4:**
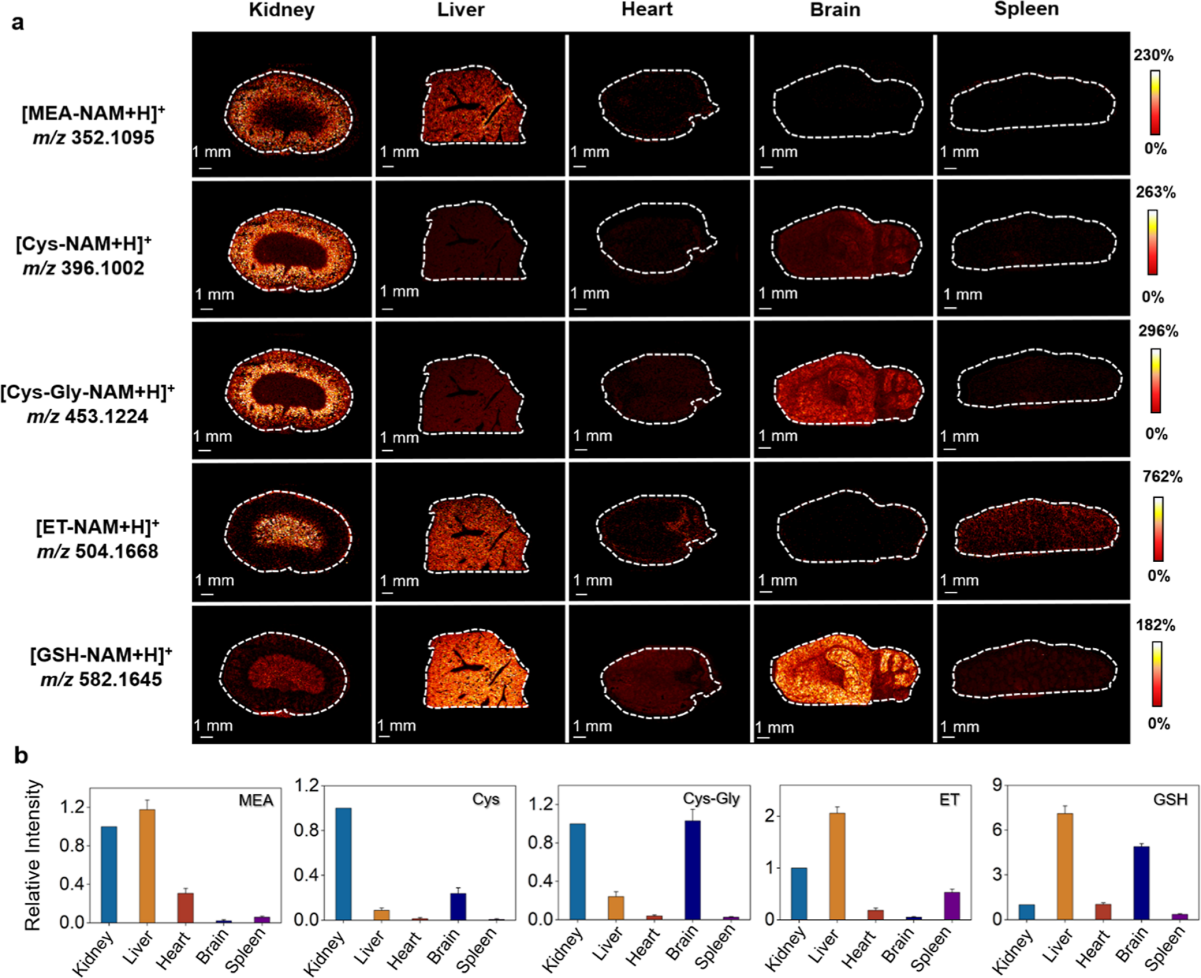
(a) Spatial distribution of different thiols in diverse tissues including kidney, liver, heart, brain, and spleen. (b) The relative intensity of thiols in different tissues. Relative intensity is defined as the ratio of the intensity of products in various tissues to that observed in the kidney (the relative intensity of products in kidney is assigned to 1.0). The relative intensity for the quantitative comparison was the mean relative intensity of three biological replicates, and the error bars indicated SD in three replicates.

Furthermore, the identification of the products was confirmed by contrasting the MS/MS spectra from tissue samples with those of the derivatized standards. As shown in Figure S15, with the exception of MEA and Cys–Gly, tissue-derived products demonstrated analogous fragmentation of the NAM probe at *m*/*z* 275.0826 and exhibited fragmentation patterns consistent with authentic standards. For the derivatized products of MEA and Cys–Gly, the distinct fragment peaks at *m*/*z* 195.0914 and *m*/*z* 259.0394 were observed, respectively, originating from collision-induced dissociation of the NAM and Cys–Gly moiety. Crucially, the fragmentation profiles of these derivatives in tissue matrices aligned with those of authentic standards, thereby confirming the authenticity of the product signal within tissues.

### Reactive Interface-Based Decoding of PFOS-Induced Redox Dysregulation in Kidney

Environmental pollutants present a significant risk to human health, with oxidative stress being a well-established mechanism of their toxicity.^45,46^ Comprehensive profiling of thiol alterations facilitates deeper mechanistic insights into the oxidative stress signaling pathways induced by pollutant exposure. Here, the perfluorooctanesulfonate (PFOS)-exposed mice were selected as the model to further evaluate the utility of the reactive interface in providing a comprehensive assessment of the dysregulation in redox parameters induced by PFOS. As shown in Figure 5a, the characteristic ion of PFOS [M–H]^−^ at *m*/*z* 498.9286 was mainly observed in the outer cortex and renal pelvis regions of kidney in the exposure group. Taking advantage of the reactive interface-coupled MSI to compare the spatial distribution and concentrations of thiols under PFOS exposure, we found that different thiols presented distinct variation patterns. As illustrated in Figure 5b,c, PFOS exposure caused a substantial reduction in the levels of Cys and GSH with decreases of approximately 25.3% and 35.2%, respectively. This decline could be ascribed to the fact that PFOS exposure triggered the production of abundant ROS within cells, whereas GSH, one of the most important intracellular antioxidants, was depleted by reaction with ROS and resulted in a significant decrease. Additionally, Cys served as a precursor for GSH synthesis, also experienced a decrease due to the accelerated depletion of GSH. Specially, the increase in MEA level may represent a compensatory response to the oxidative stress by replenishing the antioxidant pool to maintain redox homeostasis (Figure 5d).

**Figure 5:**
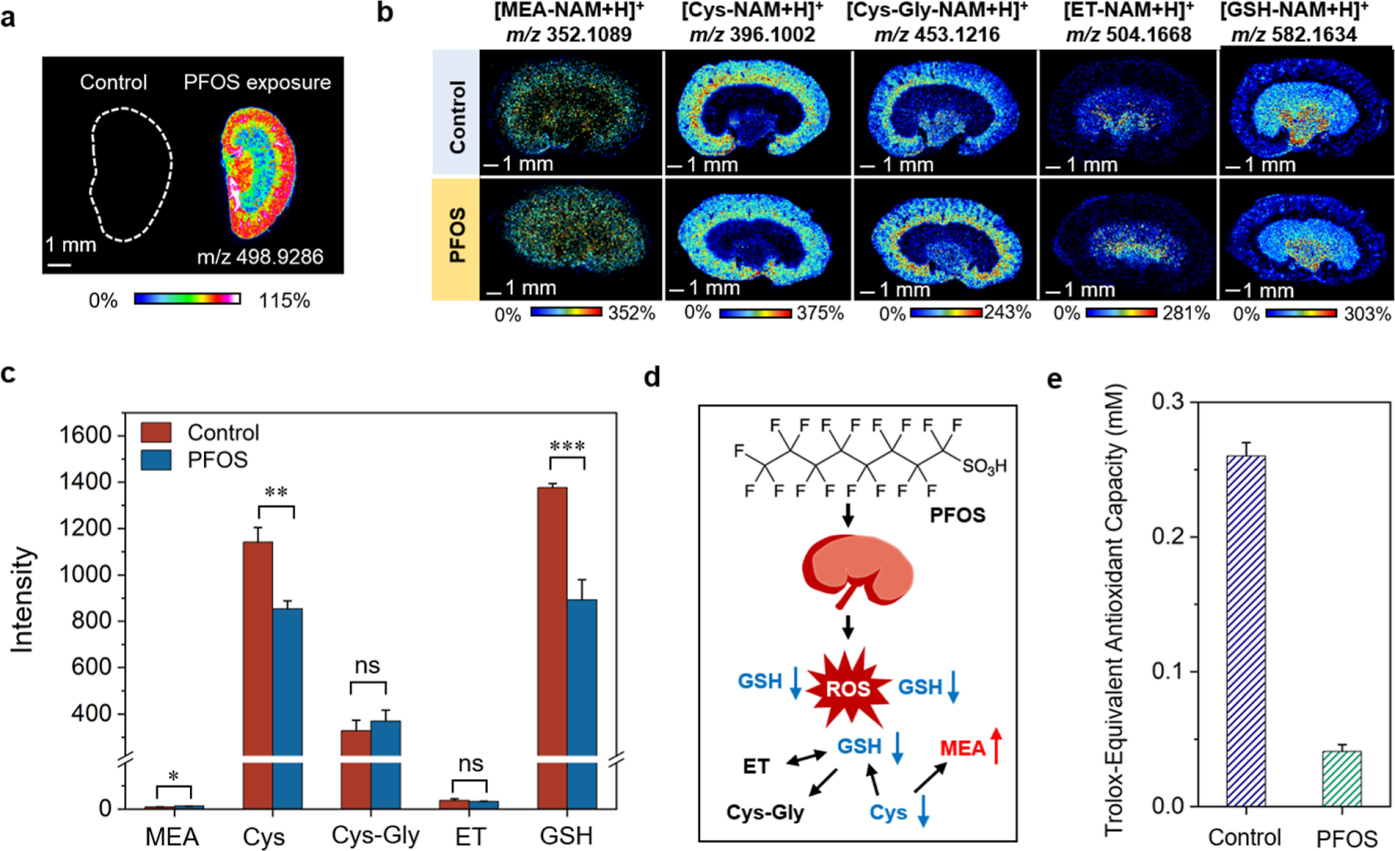
(a) Spatial distribution of PFOS in mouse kidney using the Norharmane matrix (5 mg/mL in MeOH) under the negative-ion mode. (b,c) Spatial distribution and the intensity of different thiols in kidney before and after exposure of PFOS (3 μg/g of body weight (bw)/day). Statistical analysis: *t*-test (*p < 0.05; **p < 0.01; ***p < 0.001; ns: not significant). The intensity and error bar represented the mean intensity of three biological replicates and related SD. The intensity of products was the whole-section mean intensity across the entire tissue. (d) Schematic illustration of the fluctuant thiols in response to PFOS exposure. (e) The variation of antioxidant capacity of kidney lysates quantified by the commercial assay kits (*n* = 3).

To further validate the reduction of antioxidants in tissues after PFOS exposure, we evaluated the variation of thiols in kidney lysates after PFOS exposure. As shown in Figure S16, exposure to PFOS predominantly resulted in diminished concentrations of Cys and GSH, in agreement with our MSI outcomes. In addition, we also profiled the total antioxidant capacity of kidney lysates using commercially available assay kits. As depicted in Figures 5e, S17, and S19, exposure to PFOS resulted in a substantial decrease in total sulfhydryl compounds as well as antioxidant capacity of kidney. This consistency underscores the reliability of the proposed method in evaluating pollutant-induced alterations in thiol patterns. Complementary analysis of cellular ROS enhancement and sulfhydryl compounds decrease confirmed the toxicological effects of PFOS exposure likely originated from the redox imbalance (Figures S18 and S20). Herein, our method demonstrated substantial potential in elucidating the molecular dynamics of redox networks and advancing in-depth research into the toxicological mechanisms of pollutants.

## Conclusions

In summary, we proposed a novel derivatization methodology using a NAM-precoated reactive interface for profiling the distributions of thiols by MALDI MSI, which facilitated the inquiry of thiols that was unattainable through traditional derivatization methods. The precharged acridinyl fragment of NAM probe boosted the ionization efficiency of products, while its maleimide moiety facilitated efficient recognition with thiols. Moreover, the locally high NAM concentrations in our reactive interface promoted the reaction efficiency, and the sandwich architecture minimized ion suppression of NAM probes and eliminated interaction with the matrix, allowing for simultaneous imaging of multiple thiols with enhanced sensitivity. Our approach has been utilized to assess the distribution of multiple thiols in various tissues including brain, liver, kidney, heart, and spleen, revealing differential distribution patterns potentially linked to tissue-specific physiological roles. Furthermore, the redox network in the kidney following PFOS exposure was evaluated by profiling the alterations of thiol profiles. This reactive interface-assisted derivatization strategy advances the performance of mass spectrometry derivatization imaging and provides a robust platform for exploring redox networks of diseases and pollutant-induced toxicological mechanisms.

## Supplementary Material



## Notes

Detailed experimental methods, characterization of NAM probes and reactive interface, specificity assay of NAM probes, comparison of RICD and OTCD, optimization of the experimental conditions, comparison in profiling of thiols with traditional MSI, characterization of DHB crystallization, comparable results between segmentation regions and MSI data, identification of different adducts of products, assessment of thiols variation in kidney lysates, cellular reactive oxygen assay, MS/MS spectra of the products, and commercial assay kit for quantifying total antioxidant capacity and free sulfhydryl compounds (PDF)
