# Supplementary material for: Engineered Reactive Interfaces Enable Mass Spectrometry Imaging of Multiple Thiols for Decoding PFOS-Induced Redox Dysregulation
Source: Anal Chem. 2025 Dec 29;98(1):884–93. doi: 10.1021/acs.analchem.5c05993 (PMC12809638; doi:10.1021/acs.analchem.5c05993)
Supplement: Supplementary file 1 [file ac5c05993_si_001.pdf]

Supporting information

Engineered Reactive interfaces Enable Mass Spectrometry Imaging of Multiple Thiols for Decoding PFOS-Induced Redox Dysregulation

Hongmei Xu,[1,2] Thomas Ka-Yam LAM,[2] Simin Zhang,[2] Lei Guo,[2] Chris Kong Chu Wong,[4] Chuan Dong [1,*] and Zongwei Cai [2,3,*]

[1] Institute of Environmental Science, Shanxi University, Taiyuan 030006, China

[2] State Key Laboratory of Environmental and Biological Analysis, Department of Chemistry, Hong Kong Baptist University, Hong Kong SAR, China

[3] Eastern Institute of Technology, Ningbo, China

[4] Croucher Institute for Environmental Sciences, Department of Biology, Hong Kong Baptist University, Hong Kong SAR, China

*** Corresponding author**

Phone & Fax: +852-34117348; E-mail zwcai@eitech.edu.cn; dc@sxu.edu.cn

Table of contents:

S-3, S-4 Experimental section

S-5 UV-Vis absorption spectrum of NAM probes

S-6 Specificity assay of NAM probes

S-7 NAM probes as the matrix

S-8 Comparation of detention sensitivity of metabolites by OTCD and RICD

S-9 Comparison of the utility of NAM probes in traditional OTCD and our method

S-10 Characterization of reactive interface

S-11 DHB crystallization with our method and traditional OTCD method

S-12 The optimization of tissue slice thickness

S-13 The optimization of NAM dosage for construction of reactive array

S-14 The optimization of incubation time

S-15 The consistency between segmentation results and MSI data

S-16 Spatial distribution of derivatized products with different adduct ions

S-17 Spatial distribution of thiols using traditional MSI method

S-18 Different adducts of product [GSH-NAM] in various tissues

S-19 MS/MS spectra of the products

S-20 Assessment of thiols variation in kidney lysates after PFOS exposure

S-21 Commercial assay kit for assessment of total antioxidant capability

S-22, S-23 Commercial assay kit for assessment of free sulfhydryl compounds in SV40 MES13 cells and kidney lysates

S-24 Cellular reactive oxygen assay

S-25 Table S1 The Pearson correlation coefficient between segmentation regions and MSI results

S-26 Derivatized products in mouse kidney sections

Experimental section

Reagents and Materials

MEA, ET and reserpine were purchased from Aladdin. Other standard thiols including GSH, Cys, Cys-Gly, lysine, arginine and threonine were supplied by Sigma Aldrich. NAM were obtained from TCI. Formic acid (FA), acetonitrile (ACN, HPLC grade) and methanol (MeOH, HPLC grade) were purchased from Merck (Darmstadt, Germany). 2,5-Dihydroxybenzoic acid (DHB, ≥ 98%), Norharmane (Nor) and Perfluorooctanesulfonate (PFOS, 98% purity) were purchased from Sigma Aldrich. Reactive Oxygen Species Assay Kit, Free Sulfhydryl Assay Kit with DTNB and Total Antioxidant Capacity Assay Kit with ABTS method were obtained from Beyotime.

Tissue sample preparation and quantification of thiols in kidney extracts

C57BL/6 male mice (8-10 weeks) were obtained from the Laboratory Animal Service Centre of the Chinese University of Hong Kong. Mice were housed at room temperature of 22 °C and 12 h of light/dark cycle with free access to food and water. Perfluorooctane sulfonate (PFOS, Sigma-Aldrich, 98%) was dissolved in dimethyl sulfoxide before mixing with corn oil. Mice were randomly divided control and treatment groups (n=3). The control group only received corn oil and in the exposure group, 0.3 and 3 μg/g of body weight (bw)/day of PFOS in corn oil were given for 21 days by oral gavage. Mice were sacrificed by cervical dislocations. Tissues were collected and stored at -80°C. A guideline and regulation approved by Hong Kong Baptist University's animal ethics committee were followed. All animal experiments conducted in this study was approved by the animal ethics committee (REC/22-23/0468) of Hong Kong Baptist University.

Tissue extracts were obtained as follows: kidney samples (10 mg) were mixed with 600 μL of pre-cooled 80% MeOH solution and then broken with tissue homogenizer. After centrifugation at 20000 g with 15 min, the supernatant was transferred to the vacuum concentrator for spin drying and redissolved in 100 μL of 50% MeOH solution. After centrifugation at 20000 g with 15 min, the supernatant was collected at 4 ℃ for further use. The quantification of thiol concentration in tissue

extracts was performed by plotting intensity ratios (using reserpine as the internal standard) onto the standard curves.

Cell culture and ROS assessment after PFOS exposure

The SV40 MES13 cells were cultured as follows: SV40 MES13 cells were cultured in Dulbecco's modified Eagle's medium (DMEM) supplemented with 80 U mL^{-1} of penicillin, 80 mg mL^{-1} streptomycin, 0.584 g L^{-1} l-glutamine, and 4.5 g L^{-1} D-glucose. All the media were supplemented with 10% fetal bovine serum (FBS) and cultured in a chamber with 5 % CO_2 at 37 °C. PFOS was dissolved with DMSO and cells were treated with 20 μM and 80 μM PFOS, or 0.1% DMSO for 48 h (n=3, per individual treatment group). To verify the PFOS-induced oxidative stress, the cellular ROS under different PFOS exposure (0, 20 μM and 80 μM) was quantified using 2,7-dichlorofluorescein diacetate (DCFH-DA) probes. After exposure of PFOS, the cells were washed with PBS three times and incubated with 10 μM DCFH-DA for 20 min at 37 ℃. The cells were washed twice with PBS and the fluorescence intensity was measured using laser confocal microscopy (LSM-880) through FITC channel.

As for the quantification of total thiol contents in cells, cells were cultured in six-well plates and then lysed by adding 100 μL of cell lysis buffer (P0013, Beyotime) per plate. After centrifugation at 20000 g with 15 min, the supernatant was collected at 4 ℃ for thiols quantification by using Free Sulfhydryl Assay Kit with DTNB.

UV-Vis absorption spectrum of NAM probes

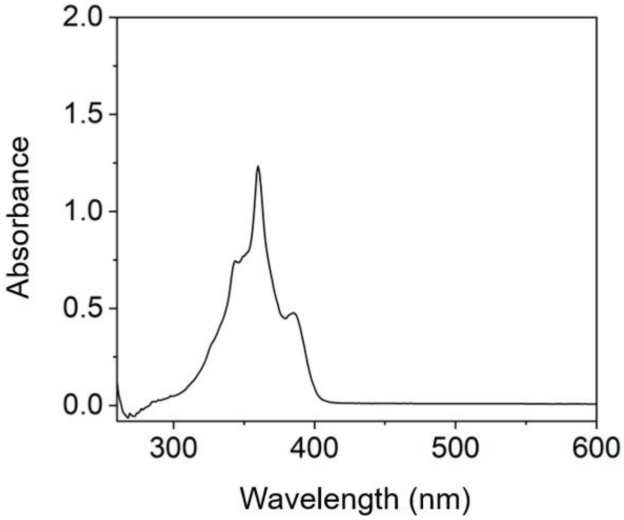

Figure S1 UV-Vis absorption spectrum of NAM probes.

Specificity assay of NAM probes

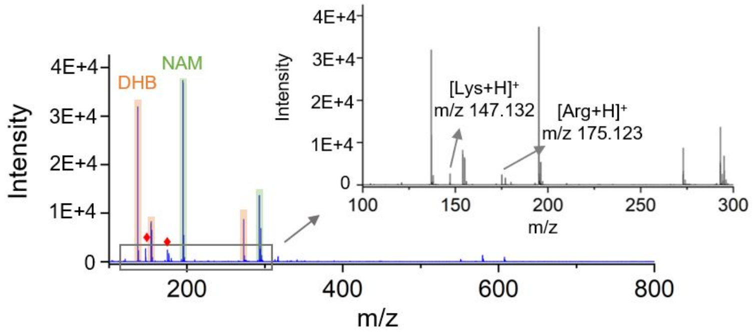

Figure S2 The MALDI MS spectra of products by incubating the NAM probes with Lys and Arg.

NAM probes as the matrix

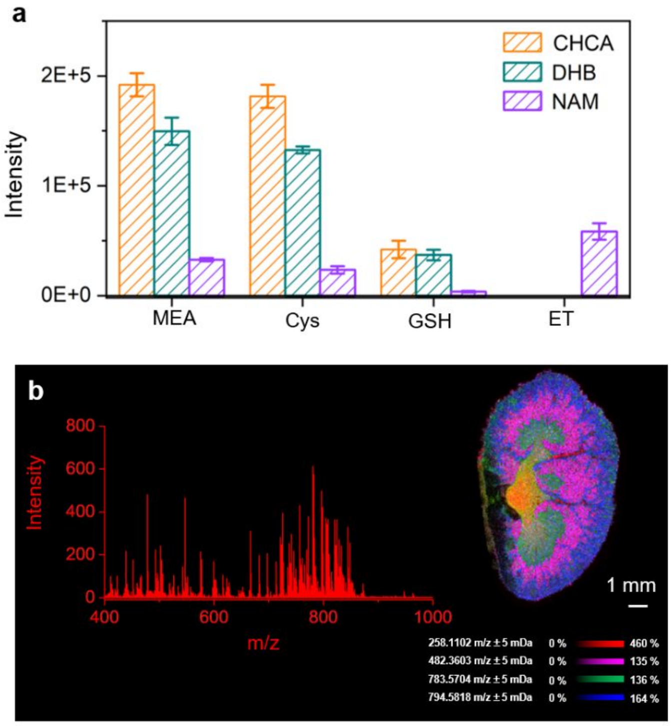

Figure S3 (a) The differentiated intensity of products with the addition of different matrices, DHB (20 mg/mL in 70% ACN, 1% H_3PO_4), CHCA (5 mg/mL in 50% ACN, 0.1% FA) and NAM (4 mg/mL in 70% ACN, 0.1% FA). (b) NAM as the matrix for mapping of metabolites in mouse kidney. Representative mass spectrum and distribution profiles of several molecules were presented here.

Comparation of detention sensitivity of metabolites in kidney

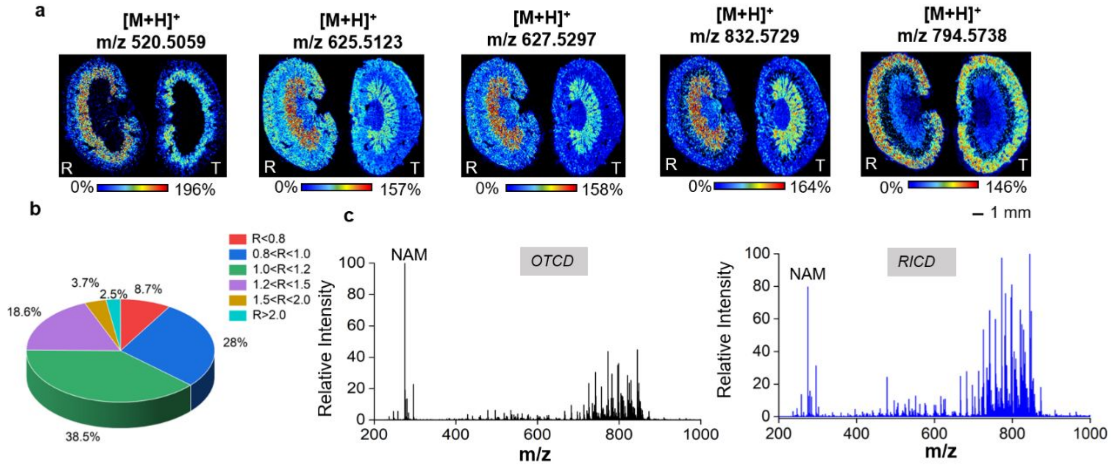

Figure S4 (a) Spatial distribution of representative metabolites in kidney using traditional derivatization method and reactive interface. (b) Statistical plot of intensity ratios of metabolites in kidney imaged by traditional derivatization method versus our method. R: RICD, T: traditional OTCD method. (c) MALDI MS spectra of kidney obtained from traditional derivatization method and our method.

Comparison of the utility of NAM probes in OTCD and our method

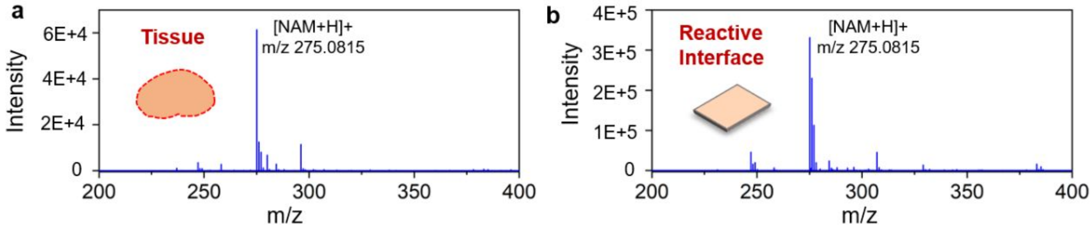

Figure S5 The MALDI MS spectra of NAM probes in kidney derivatized with NAM probes (a) and in our reactive interface (b).

Characterization of reactive interface

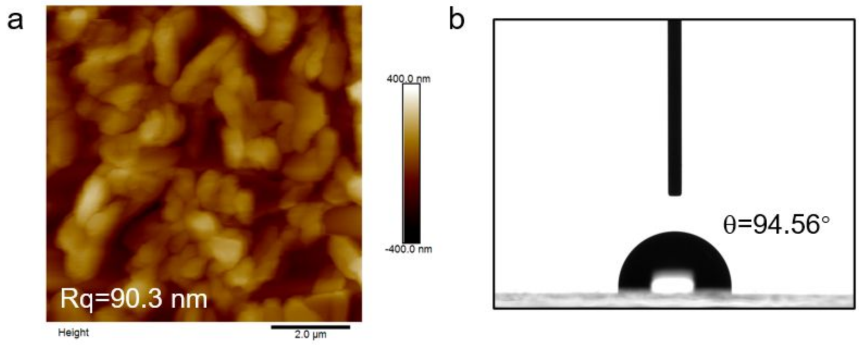

Figure S6 The AFM image (a) and contact angle measurement of water on the surface of reactive interface (b).

DHB crystallization with our method and traditional OTCD method

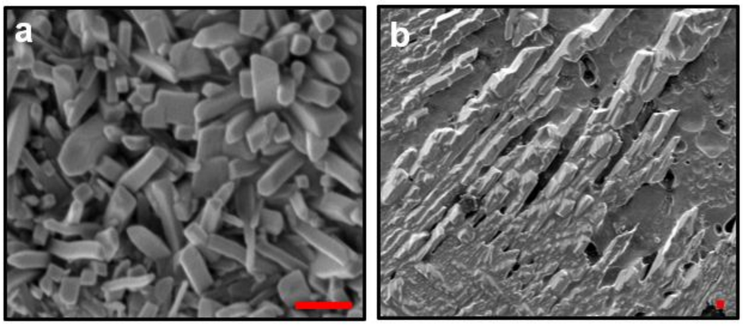

Figure S7 The SEM images of deposited DHB on the surface of tissues using our RICD method (a) and traditional OTCD method (b). Scale bar: 1μm.

The optimization of tissue slice thickness

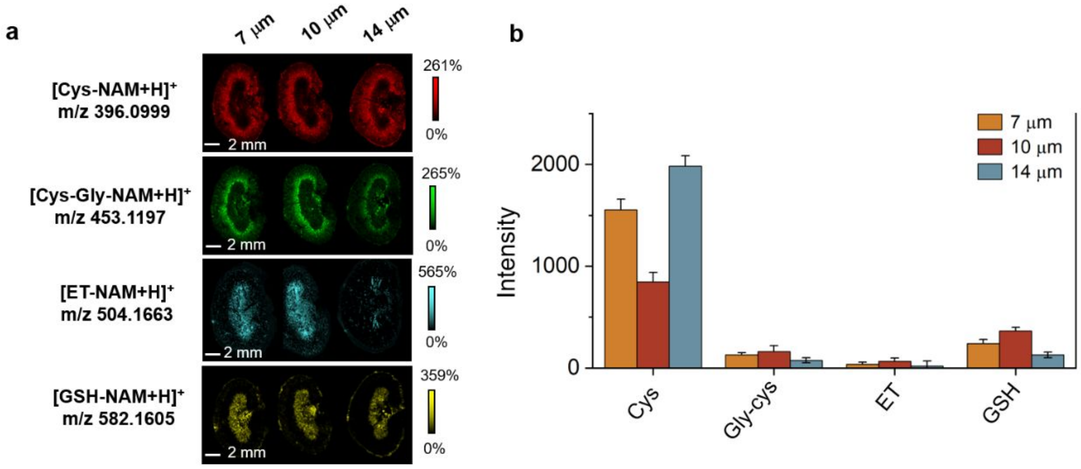

Figure S8 (a) Reactive interface-based spatial distribution of thiols in kidney sections with different thicknesses. (b) The MS intensity of products in kidney after derivatized by reactive interface. The intensity for the quantitative comparison was the mean intensity of three biological replicates and the error bars were the S in three replicates.

The optimization of NAM dosage for construction of reactive interface

The dosage of NAM probes was quantified as follows: Taking the spraying of 10 passes as an example, we prepared the NAM solution (5 mM in 70% ACN) and sprayed it at a flow rate of 30 μL/min. The time required to spray one pass was 106 s. Therefore, the total NAM deposited on the ITO after 10 spraying cycle is: 30 μL/min × 106 s × 10 × 5 mM = 2.65×10^{-6} mol. The dimension of the ITO used are 7.5 cm × 2.5 cm, resulting in the deposition amount of 0.039 mg/cm² (The molecular weight of the NAM probe is 274.0736).

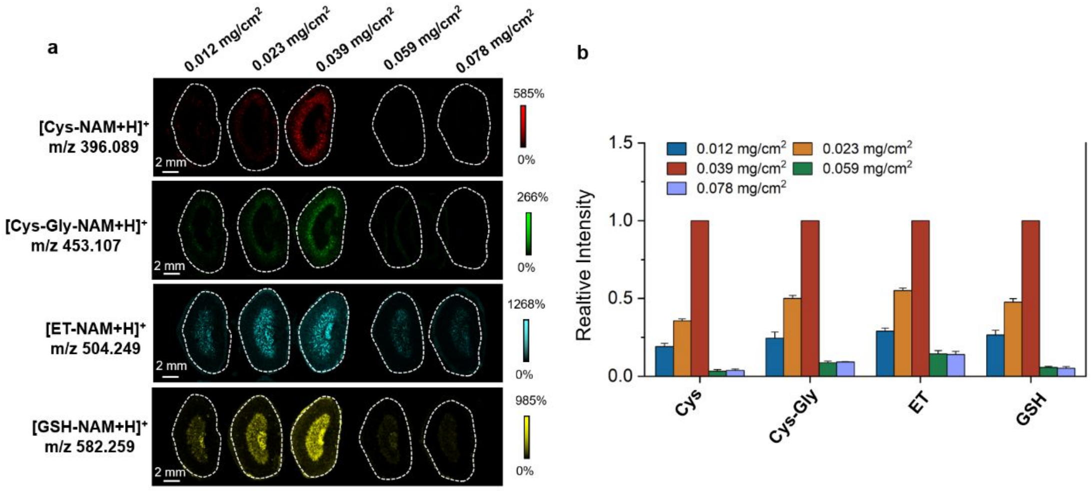

Figure S9 (a) Spatial distribution of thiols in kidney via reactive interface pre-coated with different dosage of NAM probes. (b) The relative MS intensity of products in kidney after derivatized by reactive interface. The intensity of products derivatized by reactive interface with spraying of 10 pass as the reference (assigned value = 1.0). The relative intensity for the quantitative comparison was the mean relative intensity of three biological replicates and the error bars indicate SD in three replicates.

The optimization of incubation time

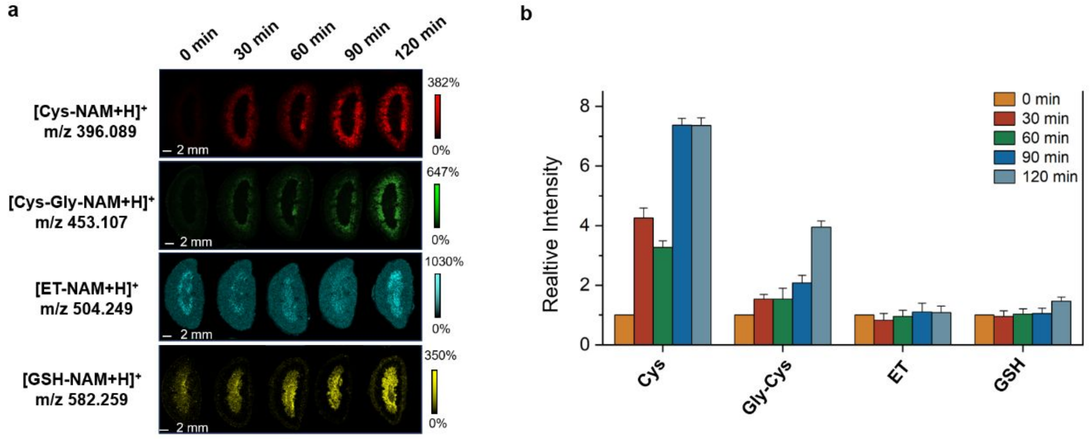

Figure S10 (a) Spatial distribution of thiols in kidney using reactive interface under different incubation time in a humidified environment. (b) The relative MS intensity of products in kidney derivatized by reactive interface. The intensity of products derivatized by reactive interface without incubation as the reference (assigned value = 1.0). The relative intensity for the quantitative comparison was the mean relative intensity of three biological replicates and the error bars indicate SD in three replicates.

The consistency between segmentation results and MSI data

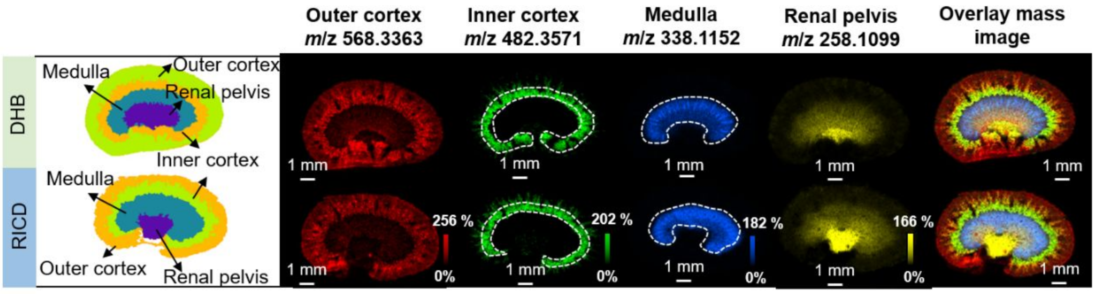

Figure S11 Color-encoded segmentation results and the representative distribution of metabolites in kidney obtained by our method and traditional MSI.

Spatial distribution of derivatized products with different adduct ions

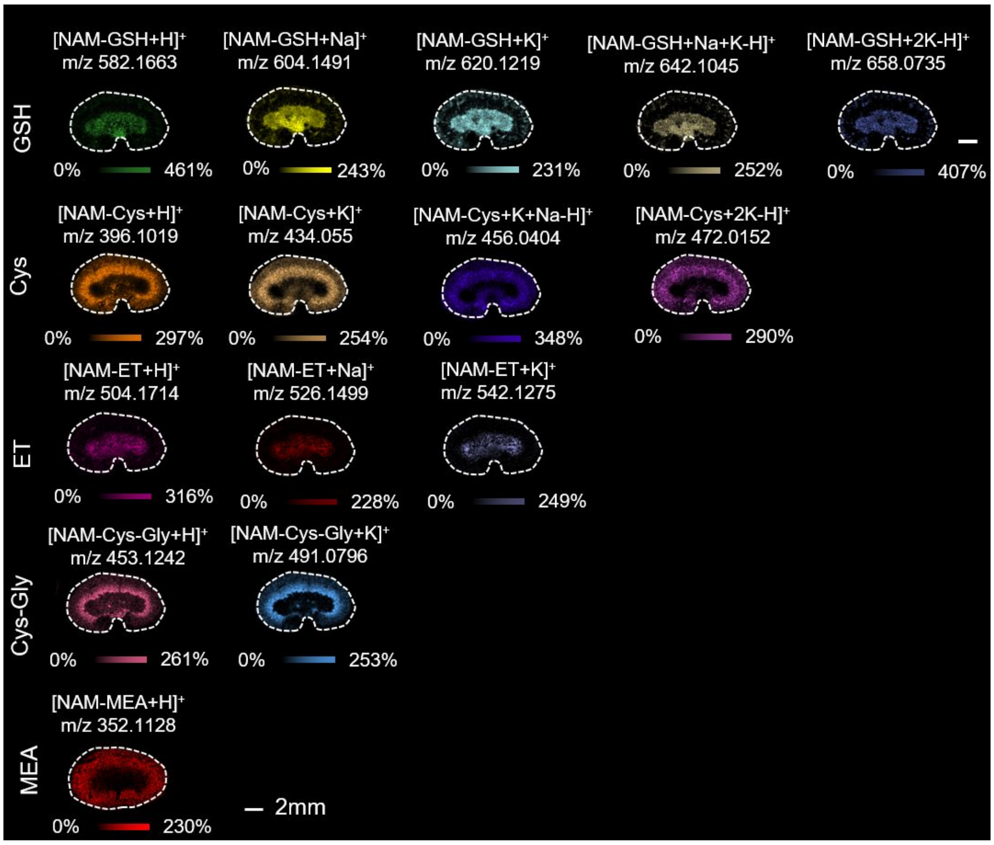

Figure S12 Spatial distribution of derivatized products in kidney using reactive interface.

Spatial distribution of thiols using traditional MSI method

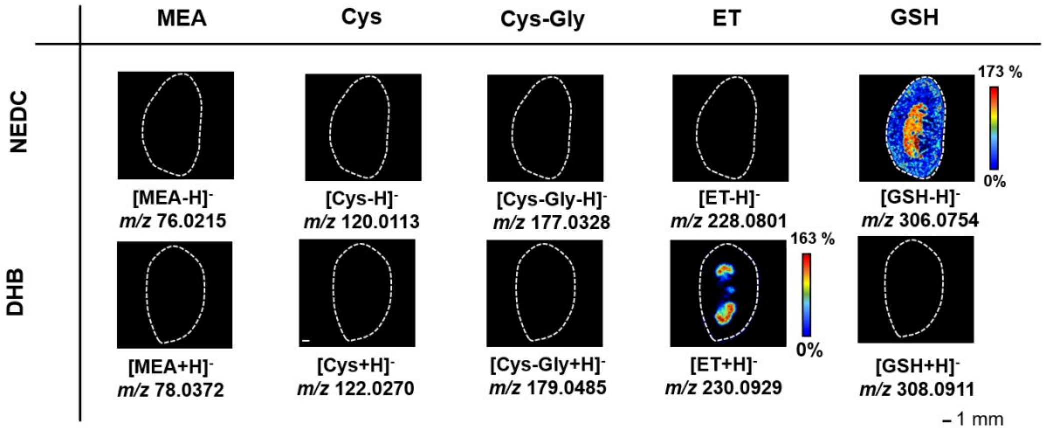

Figure S13 Spatial distribution of thiols using traditional MSI method by adopting NEDC and DHB as the matrix.

Different adducts of product [GSH-NAM] in various tissues

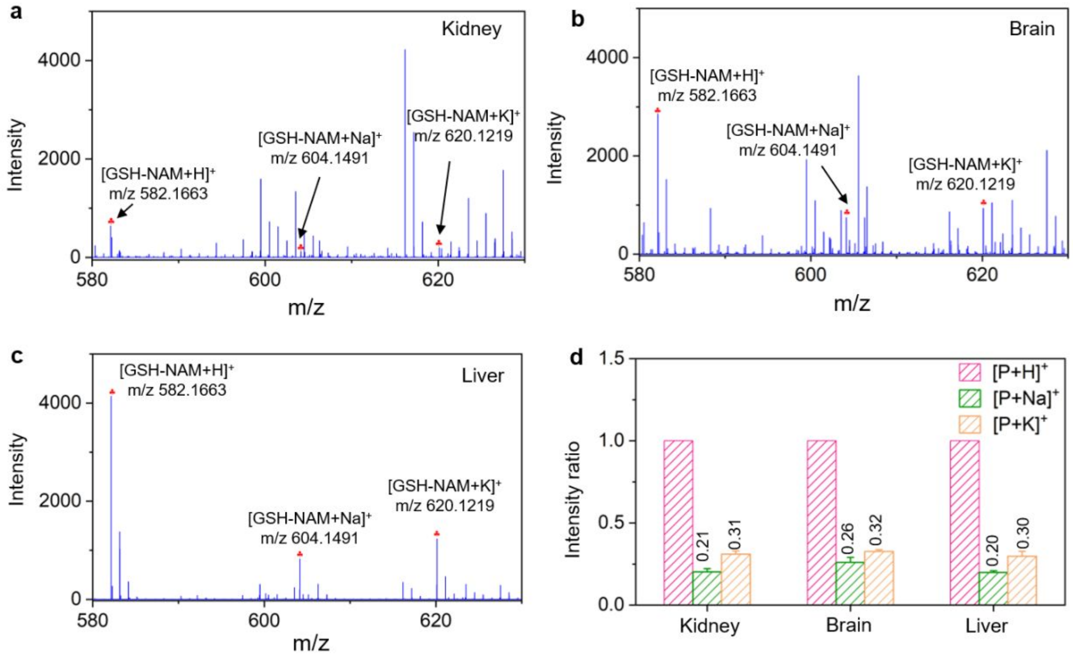

Figure S14 (a-c) MALDI MS spectra of different adducts of [GSH-NAM] product in kidney (a), brain (b) and liver (c). (d) The intensity ratio of different adducts of [GSH-NAM] in different tissues. The intensity of protonated peak $[M+H]^+$ was selected as the reference (assigned value = 1.0). The error bars indicate SD in three replicates.

MS/MS spectra of the products

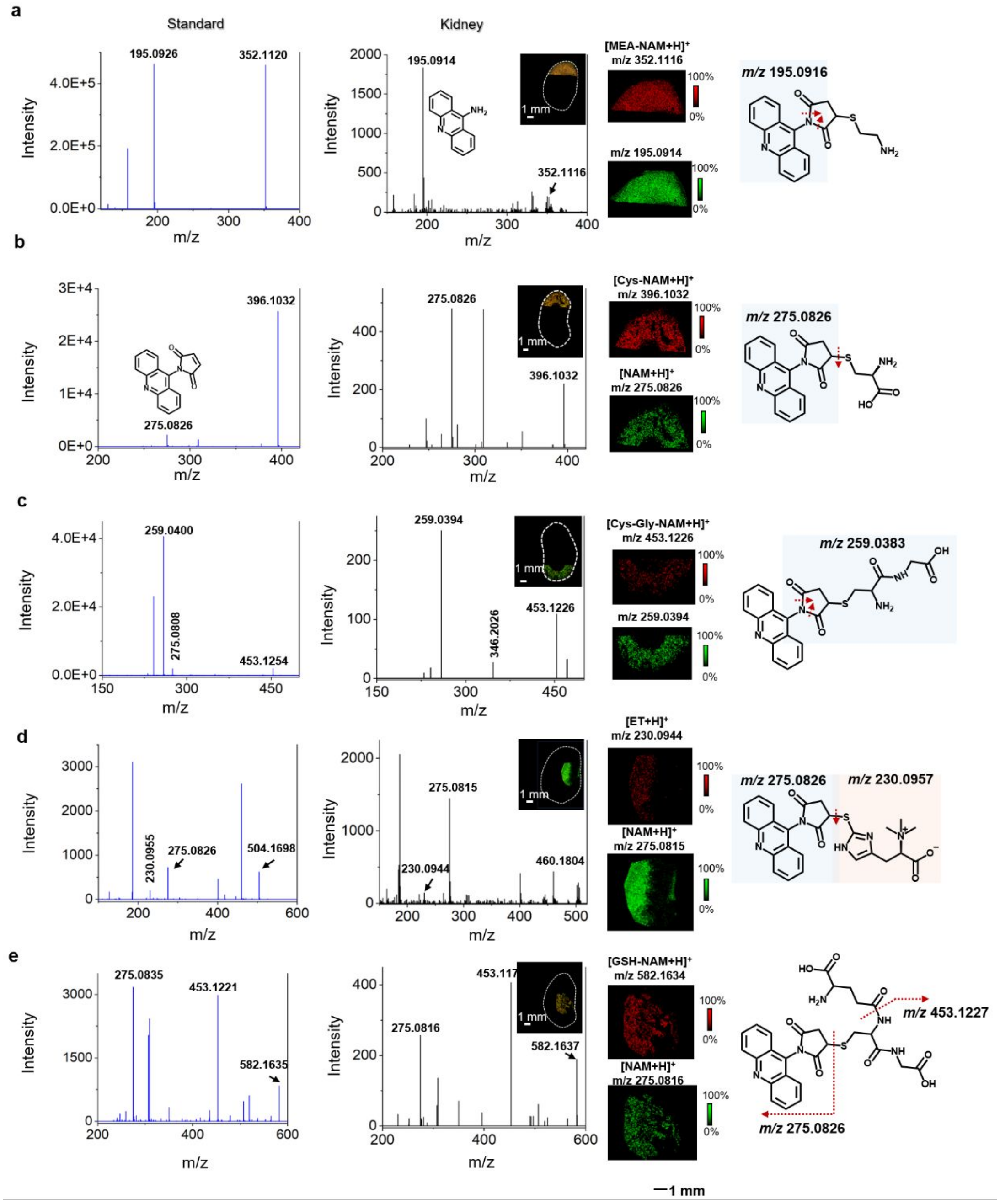

Figure S15 MS/MS spectra of derivatized products in kidney and authentic standards. (a) MEA-NAM adduct. (b) Cys-NAM adduct. (c) Cys-Gly-NAM adduct. (d) ET-NAM adduct. (e) GSH-NAM adduct.

Assessment of thiols variation in kidney lysates after PFOS exposure

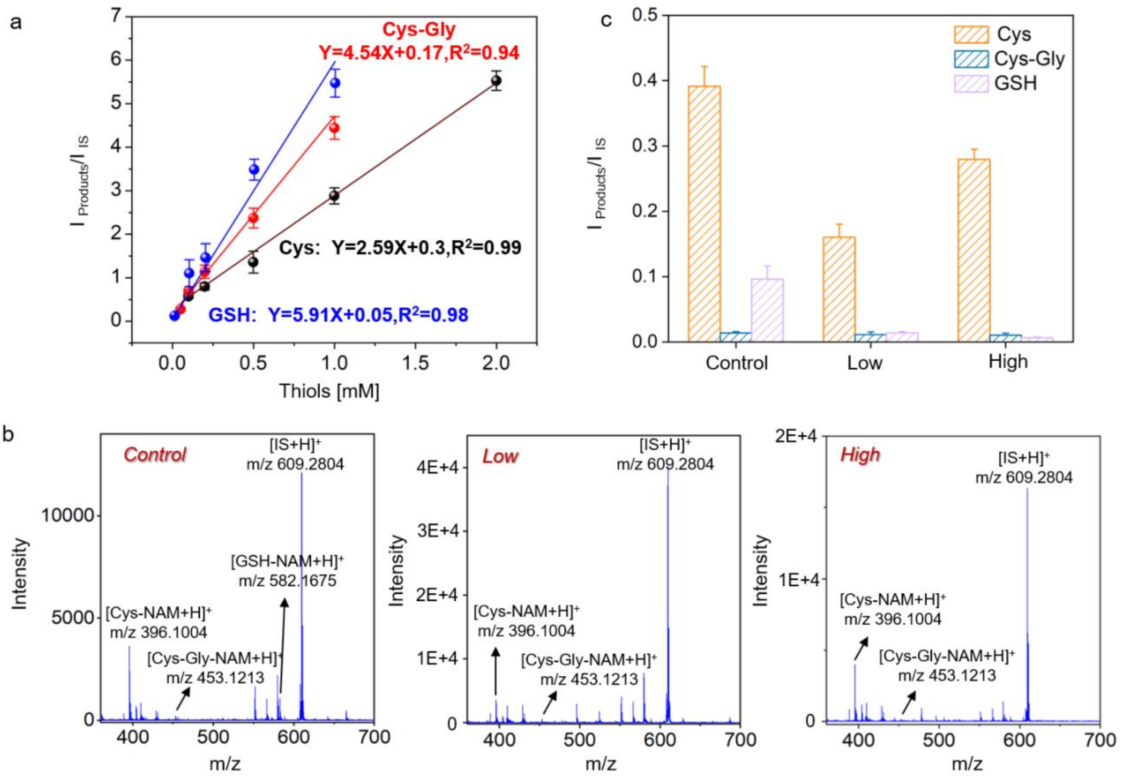

Figure S16 (a) Relationship between the intensity ratios of products and the concentrations of the GSH, Cys-Gly and Cys. MALDI MS spectra (b) and corresponding intensity ratios of products (c) in kidney lysates under exposure of PFOS with different concentrations (n=3). Control: no PFOS exposure, Low: 0.3 μg/g of body weight/ day of PFOS, High: 3 μg/g of body weight/ day of PFOS. The reserpine was selected as the internal standard (*m/z* 609.2806). $I_{Product}$ and I_{IS} represented MS peak intensity of products and IS.

Commercial assay kit for assessment of total antioxidant capability of kidney lysates after PFOS exposure

The total antioxidant capacity in kidney lysate was referenced to Trolox. Therefore, by virtue of the rapid ABTS method, a linear curve of the concentrations of Trolox versus relative absorbance of oxidative ABTS ($ABTS^{\cdot+}$) at 414 nm was plotted. The total antioxidant capability of kidney lysates was calculated by measuring the relative absorbance of $ABTS^{\cdot+}$ referred to the linear curve.

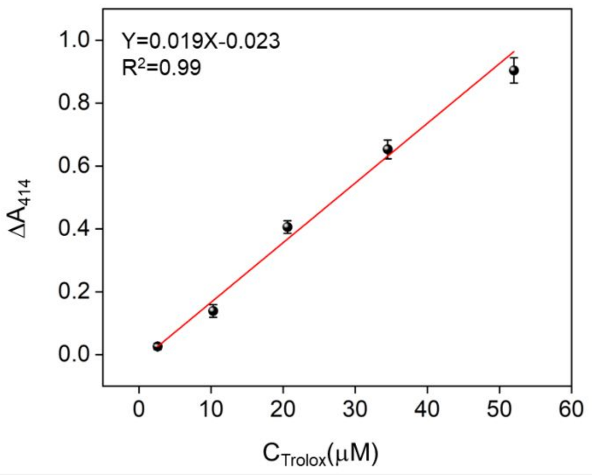

Figure S17 The relationship between the relative absorbance of oxidative ABTS ($ABTS^{\cdot+}$) at 414 nm and the concentrations of Trolox.

Commercial assay kit for assessment of free sulfhydryl compounds within SV40 MES13 cells after PFOS exposure

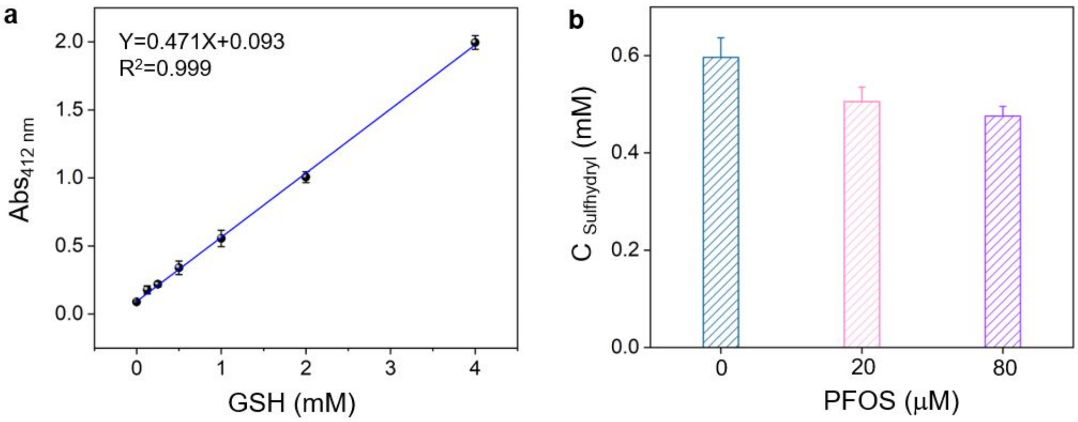

Figure S18 (a) The linear relationship of the concentration of GSH and the absorbance of TNB $^{2-}$ at 412 nm. (b) The total sulfhydryl concentration within SV40 MES13 cells under exposure of PFOS with different concentrations (n=3).

Commercial assay kit for assessment of free sulfhydryl compounds of kidney lysates after PFOS exposure

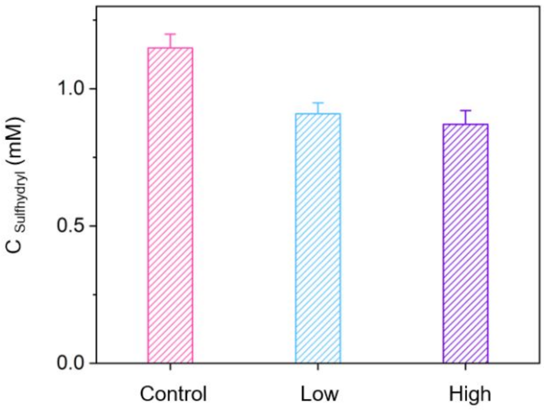

Figure S19 The total concentrations of sulfhydryl compounds in kidney lysates under exposure of PFOS with different concentrations (n=3).

Cellular reactive oxygen assay

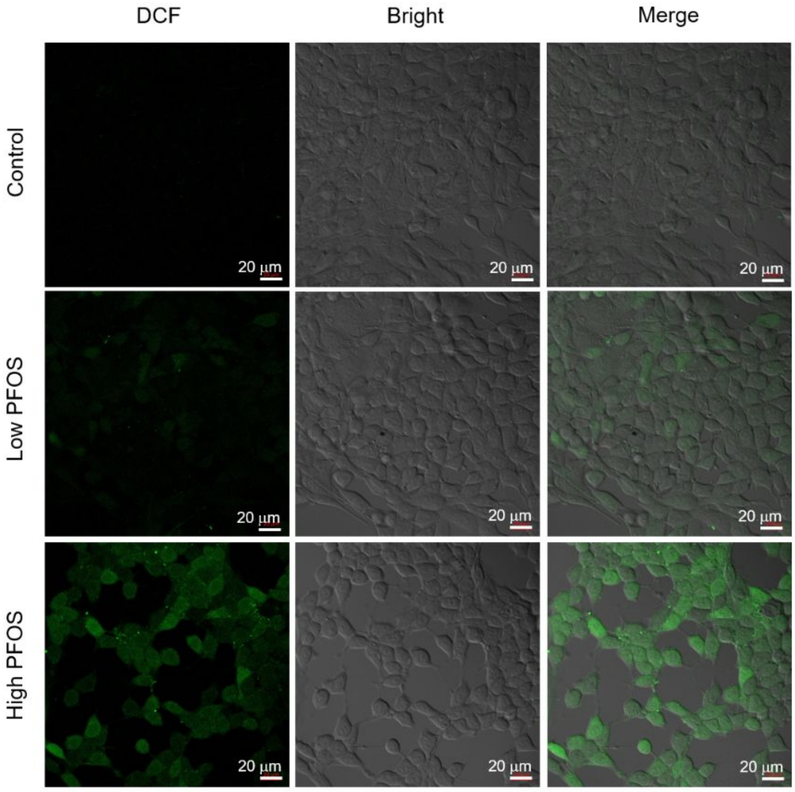

Figure S20 CLSM images of SV40 MES13 cells after exposure of different concentrations of PFOS for 48 h (control: 0 μM, low PFOS: 20 μM and high PFOS: 80 μM). The cellular ROS was profiled by DCFH-DA probes using FTIC channel.

Table S1 The Pearson correlation coefficient between segmentation regions and MSI results

m/z	DHB	RICD
258.1099	r=0.82 (p-value < 0.05)	r=0.78 (p-value < 0.05)
338.1152	r=0.81 (p-value < 0.05)	r=0.93 (p-value < 0.05)
482.3571	r=0.78 (p-value < 0.05)	r=0.77 (p-value < 0.05)
568.3363	r=0.69 (p-value < 0.05)	r=0.76 (p-value < 0.05)

r: Pearson correlation coefficient

By calculating the correlation between the segmentary sub-regions and the distribution patterns of metabolites in two MSI data and comparing their consistency, we can confirm the accuracy of our method with traditional MSI. As shown in Table S1, most representative metabolites presented high Pearson correlation coefficient with segmentation regions, thereby confirming the precise imaging distribution profiles using our methods.

Tabel S2 Derivatized products in mouse kidney sections

**Products**	**Molecular Formula**	**Ion type**	**Exp. *m/z***	**Cal. *m/z***	**Delta (ppm)**
MEA-NAM	$C_{19}H_{17}N_3O_2S$	M+H	352.1128	352.1114	3.9
Cys-NAM	$C_{20}H_{17}N_3O_4S$	M+H	396.1019	396.1012	1.7
		M+K	434.055	434.0571	4.8
		M+K+Na-H	456.0404	456.0391	2.8
		M+2K-H	472.0152	472.0130	4.6
Cys-Gly-NAM	$C_{22}H_{20}N_4O_5S$	M+H	453.1242	453.1227	3.3
		M+K	491.0796	491.0786	2.0
ET-NAM	$C_{26}H_{25}N_5O_4S$	M+H	504.1714	504.1700	2.8
		M+Na	526.1499	526.1519	3.8
		M+K	542.1275	542.1259	2.9
GSH-NAM	$C_{27}H_{27}N_5O_8S$	M+H	582.1663	582.1653	1.7
		M+Na	604.1491	604.1472	3.1
		M+K	620.1219	620.1212	1.1
		M+Na+K-H	642.1045	642.1031	2.2
		M+2K-H	658.0735	658.0770	5.3
